# Guideline documents on caesarean section on maternal request in Sweden: varying usability with a restrictive approach

**DOI:** 10.1186/s12913-023-10077-7

**Published:** 2023-10-18

**Authors:** Magdalena Mattebo, Inger K Holmström, Anna T Höglund, Mio Fredriksson

**Affiliations:** 1 School of Health, Care and Social Welfare, Mälardalen University, Västerås, Sweden; 2 Department of Public Health and Caring Sciences, Health Services Research, Uppsala University, Uppsala, Sweden; 3 Centre for Research Ethics & Bioethics (CRB), Department of Public Health and Caring Sciences, Uppsala University, Uppsala, Sweden

**Keywords:** Caesarean section on maternal request, CSMR, The framework method, Guidelines, Reproductive autonomy

## Abstract

**Background:**

Globally, studies illustrate different approaches among health care professionals to decision making about caesarean section (CS) and that attitudes regarding the extent to which a CS on maternal request (CSMR) can be granted vary significantly, both between professionals and countries. Absence of proper regulatory frameworks is one potential explanation for high CSMR rates in some countries, but overall, it is unclear how recommendations and guidelines on CSMR relate to CSMR rates. In Sweden, CSMR rates are low by international comparison, but statistics show that the extent to which maternity clinics perform CSMR vary among Sweden’s 21 self-governing regions. These regions are responsible for funding and delivery of healthcare, while national guidelines provide guidance for the professions throughout the country; however, they are not mandatory. To further understand considerations for CSMR requests and existing practice variations, the aim was to analyse guideline documents on CSMR at all local maternity clinics in Sweden.

**Methods:**

All 43 maternity clinics in Sweden were contacted and asked for any guideline documents regarding CSMR. All clinics replied, enabling a total investigation. We used a combined deductive and inductive design, using *the framework method for the analysis of qualitative data in multi-disciplinary health research*.

**Results:**

Overall, 32 maternity clinics reported guideline documents and 11 denied having any. Among those reporting no guideline documents, one referred to using national guideline document. Based on the Framework method, four theme categories were identified: CSMR is treated as a matter of fear of birth (FOB); How important factors are weighted in the decision-making is unclear; Birth contracts are offered in some regions; and The post-partum care is related to FOB rather than CSMR.

**Conclusion:**

In order to offer women who request CS equal and just care, there is a pressing need to either implement current national guideline document at all maternity clinics or rewrite the guideline documents to enable clinics to adopt a structured approach. The emphasis must be placed on exploring the reasons behind the request and providing unbiased information and support. Our results contribute to the ongoing discussion about CSMR and lay a foundation for further research in which professionals, as well as stakeholders and both women planning pregnancy and pregnant women, can give their views on this issue.

## Background

Several studies illustrate an increase in the rate of caesarean section (CS) [1–3], which is sometimes described as dramatic, causing a major problem [1], and not being medically justifiable [3]. The latest data from 150 countries show that 18.6% of all births occur by CS. [3]. There may be a number of reasons behind the increase in CS, e.g. CS on maternal request (CSMR), provider attitudes, health system characteristics as well as economic, organizational, social and cultural factors’ [3, 4].

It has become a controversial topic that women with low-risk pregnancies should have the right to make decisions regarding their mode of delivery [5], i.e. to be able to request a CS. A systematic review and meta-regression by Begum et al. from 2021 reported an absolute proportion of CSMR between 0.2 and 42% and a difference between 0.9 and 60% (across studies and subgroups). The authors showed that upper-middle-income countries have an 11 times higher incidence of CSMR in relation to the total number of deliveries compared to high-income countries, and that CSMR is the most common in the Middle East and East Asia [2]. A systematic review of reasons for requesting a CS showed that the most common reasons were fear of labour pain, fear of birth (FOB), fear of urinary incontinence, fear of pelvic floor and vaginal trauma, and anxiety about baby injury/death [1].

Previous studies illustrate great differences in professional opinions on CSMR. For example, 85% of obstetricians in the US and 77% in Australia would perform a CSMR, while only a respective 15 and 23% in Spain and Canada [5]. In Iran, obstetricians considered CS to be less likely to attract litigation, while also generating more income and being a convenient scheduled procedure. Furthermore, midwives have been shown to support CSMR to a lower extent; for example, only 22–23% of midwives in Sweden, who rather see as their duty to provide information, in order to support women in choosing the safest mode of delivery, support it [5]. As the debate on CSMR is not only about women’s rights, but also the demands of the healthcare system, conflicting opinions among, e.g., obstetricians may depend on whether they prioritize the free choice of the individual or see the risks involved with health service resource limitations. In healthcare systems with a higher degree of private funding, more stakeholders—particularly obstetricians—support CSMR [4, 5].

One potential explanation for the higher proportion of CSMR in upper middle-income countries, illustrated by Begum et al. [2], is that there is an absence of a proper regulatory framework, which may lead to a misuse of patient autonomy in decision-making [2]. In contrast, some organizations have clear recommendations and guidelines regarding CSMR, which often include information and counselling before it can be considered. It is unclear, however, how these guidelines are implemented and the level of adherence to them; in addition, research suggests that maternity care providers tend to recommend the mode of delivery that is consistent with their personal preference [5]. Such differences may be linked to differences in CS rates noted both between and within countries [3]. Contrary to regions such as Latin America and some European countries (e.g. Cyprus, with a CS rate of 57%), several European countries have succeeded in controlling their CS rates over time. In 2015, Finland, Iceland, and Norway had CS rates of 16–17%, and Sweden 18% [6].

In Sweden, the country in focus in this article, CSMR rates increased by 80% between 1990 and 2001 [7]. However, at present, only 5% of primiparous women give birth through a planned CS, and of these only one in four is a CSMR [8]. Among multiparous women, 9.5% give birth through a planned CS and about a third of these are at the mother’s request. Nonetheless, these figures say nothing about how many women want a CS but are denied it by the healthcare services, and ongoing debates on social media as well as traditional media suggest that this is a substantial problem, causing high levels of dissatisfaction among pregnant women [9]. A recent report [8] also pointed to a polarization between healthcare staff and women wanting a CSMR, involving a woman’s right to make decisions on the mode of delivery and how safe vaginal delivery is in comparison with CS without medical reasons.

Betrán et al. suggest that one factor associated with higher rates of vaginal births may be strict policies on CSMR [3]. According to Swedish law a woman cannot demand to have a CS [7], but there may nonetheless be reasons to accommodate a woman’s preference. National medical indications on CSMR, published in 2011 [10], contains a review of maternal and neonatal short- and long-term risks that are of importance in the discussion on CSMR (concluding that when there are no medical reasons, CS usually implies a higher risk for mother and child, both short- and long-term). They were developed by a number of national stakeholder organizations and authorities, among them the Swedish Society of Medicine and the National Board of Health and Welfare. The target groups are healthcare professionals making decisions on CSMR, clinic managers as well as healthcare decision-makers and the document is meant to serve as decision support, so that resources can be used in the best way. Furthermore, the document specifies under what conditions it is relevant to accommodate a woman’s wish for a CS without medical reasons, however conveying a restrictive approach. There are three types of conditions: (i) *patient-related* (e.g. that the reasons are assessed to be sufficiently compelling); (ii) care process-related; and (iii) care provider-related [7] (Table 1). It is concluded that a woman’s request for a CS should be accommodated if her reasons are regarded to have been sufficiently compelling (i) and she maintains her request after receiving relevant information and being offered supportive discussions or other forms of care (ii). In this article, the empirical focus is the third condition; i.e., that the maternity clinics should have local guidelines for how to provide care for women with different motives and risk levels, aiming for standardization and reflecting the local care tradition. These local guidelines affect how patient-related conditions and care-process related conditions are interpreted at the maternity wards. It should be noted, however, that neither the national medical indications nor the local guidelines are mandatory, but rather recommendations.

The outcomes of maternity and delivery care is monitored through the Swedish Pregnancy Registry covering over 98% of all registered deliveries [11]. Statistics from the Swedish National Board of Health and Welfare show that CS rates, as well as the extent to which maternity clinics consider they can meet women’s demands for CSMR, vary among Sweden’s 21 self-governing regions, which are responsible for the funding and delivery of healthcare [12]. It is also shown that local routines rather than national medical indications contribute to the great differences in CS rates. This points to the importance of clear and interpretable national guidance—not only as support for healthcare staff in making well-grounded and evidence-based decisions, but also to guard consistent decisions regardless of which healthcare staff provide care.

To further understand considerations for CSMR requests and existing practice variations, the aim here was to analyse guideline documents on CSMR at all local maternity clinics in Sweden. It should be pointed out, that there are no separate guidelines for primigravida and pregnant women with experience from previous CS. The same guidelines are used regardless. The analysis focused on the documents’ descriptions of how the procedure is carried out when a woman wants a CSMR, who makes the decision, and how the decision is made (i.e. how different reasons for CSMR are valued). Thus, this study adds to the literature on the impact of recommendations and guidelines regarding CSMR for CSMR practice.

**Table 1:** National indications for caesarean section on maternal request in Sweden

Conditions	
Patient-related conditions	• The woman has reported the reason for her request for caesarean section, and the reason is assessed to be a sufficiently compelling. A high degree of FOB could be a factor, as could acute psychiatric illness and being a victim of sexual abuse; *but not* age in itself, not previous caesarean section, not previous birth injuries such as significant tearing and incontinence (unless they are persistent), usually not a previous stillborn or injured infant related to pregnancy or delivery, and not practical reasons such as planning. • The woman adheres to her wish after receiving and understanding information about the short- and long-term consequences of different modes of birth for herself and the child, and after receiving counselling or another type of support.
Care process-related conditions	• A structured medical history should be taken and the woman’s reasons (and their assessed strength) evaluated. FOB should be graded using a psychometric tool. • A probability and risk assessment should be performed, considering ‘the probability of additional pregnancies and the likelihood that a vaginal birth would result in an emergency caesarean section, as well as the risk of any elective caesarean section’. • Information should be provided about the short- and long-term consequences of a caesarean section. It should be accurate and detailed and given both verbally and in writing, and the provider should ensure that the woman understands it. • Appropriate strategies for supporting the woman have been designed based on ‘an aggregate evaluation of the strength of the reasons women have stated and risk assessment for a possible caesarean section’. • Supportive sessions have been offered and carried out.
Care provider-related conditions	• The delivery clinics should have a policy for how to handle women with different motives and risk levels. The policy should reflect the local care tradition and aim for standardization within the clinic.

## Methods

### Design

This study was a total investigation with a combined deductive and inductive design using *the framework method for the analysis of qualitative data in multi-disciplinary health research* (the Framework Method) among maternity clinics in Sweden.

### Setting

All 43 maternity clinics were contacted by e-mail. We requested guideline documents, if there were any, about CSMR.

### Data collection

Data were collected during September-December 2021. All maternity clinics ultimately responded, even though some needed up to four reminders.

### Analysis

The Framework Method was used because it is a systematic and flexible approach to analysing qualitative data in multi-disciplinary health research [13], such as in the current study with researchers representing the fields of midwifery, nursing, care ethics, and health policy and governance. Although most often used for analysing interviews (elicited texts), the method can also be used for studying pre-existing texts such as guideline documents. It allows for comparing and contrasting data across cases, here the local guideline documents at maternity clinics in Sweden—with the aim of generating themes. The study was neither exclusively deductive or inductive; a combined approach was used as the point of departure was the study’s three focus areas: the CSMR procedure, the decision-makers, and how the decision is made. Thus, the themes were not determined beforehand but were instead guided by the three focus areas.

Stage 1 (transcription) was not applicable. During Stage 2 (familiarization), authors MM and MF read half of the guideline documents each to become familiar with the content. In the following stage (3, coding), MM and MF used open coding on half of the guideline documents each to capture relevant aspects relating to the three focus areas. It became apparent that the documents mostly covered FOB and how to handle FOB to a great extent. In Stage 4 (developing an analytical framework), authors MM and MF met and compared coding to discuss the categories and codes to be applied in the coding of all guideline documents. The analytical framework underwent two iterations before it was applied to all guideline documents in Stage 5. Consensus on the final categories was achieved among all the authors. The final categories were: CSMR is treated as a matter of FOB; How important factors are weighted in the decision-making is unclear; Birth contracts are offered in some regions; and The postpartum care is related to FOB rather than CSMR. During this stage, three guideline documents were coded by MM and MF to ensure that the analytical framework was used in the same way. In Stage 6, the data belonging to the codes and categories were summarized into a framework matrix in an ongoing discussion between authors MM and MF. A compacted matrix was also completed. Stage 7 (interpreting the data) was, in practice, undertaken throughout the analysis in discussions among all the authors.

## Results

Of all clinics, 32 reported guideline documents and 11 denied having any. Among those reporting no guideline documents, one referred to using national guideline document [10]. The documents varied between 1 and 9 pages, with 13 presenting statistics on FOB and 15 addressing how it can be measured. In 14 of the guideline documents, there were references to scientific publications. In the documents the terms *woman*, *patient*, and *pregnant person* were most used when referring to care recipients, but in some documents the terms *couple*, *parents*, and *they* were also used (Table 2).

**Table 2:** Overview table

Items	Number of clinics
Number of pages 1–2	9
Number of pages 3 or over	23
Contains scientific references	17
Handling CSMR as FOB	23
Vaginal birth stated as goal	12
Offers birth contract	13 (not included when listed as *birth planning*)
Specifies who makes the decision	20
Specifies how the decision is made	0
Point in pregnancy at which ***decision*** about CS is made	3
List of relevant factors for CS	8
List of possible factors for FOB	12

### CSMR is treated as a matter of FOB

There were several guideline documents dealing with CSMR as primarily about FOB and how to handle FOB. Typically, the guideline documents described, that women expressing severe FOB (often measured using the FOB Scale, FOBS, with a cut off at > 60) are referred to a specialized maternity clinic by the regular maternity care provider in primary care. This specialized clinic complements the regular assessment and treatment of FOB handled by midwives and physicians in primary care. Typically, the specialized clinic makes a more in-depth mapping of the woman’s FOB: why it has occurred, particular vulnerabilities, and how the preparations for giving birth can be supported. The focus is primarily on the woman’s resources and strengthening strategies for improving the sense of security related to giving birth. The woman can be referred to an obstetrician, a midwife with specialized competence in FOB, a counsellor, or a maternal and child health psychologist, and is typically offered individual support sessions, e.g. working with psychoprophylactic methods. In one of the guideline documents, it was expressed that ‘*it is a goal to support women who are afraid of childbirth in their belief in themselves and in their ability to give birth’.* In yet another guideline document it was expressed like this: *‘The focus is primarily on the pregnant person’s own resources and strengthening strategies for an increased sense of security before childbirth’.*

Most of the policy guideline documents on FOB also incorporated descriptions of how to handle CSMR. Those who request a CS without a medical reason are referred to a specialized maternity clinic (and in two guideline documents it was explicitly stated that this is mandatory and cannot be declined). For example: ‘*If the patient expresses a wish for [a CS] without a medical indication, she will immediately be referred to the Aurora clinic [supports those with FOB] irrespective of length of pregnancy. In this case, Aurora is not an offer but a requirement, and cannot be declined’*. It is clear that the first meeting at the specialized maternity clinic—whether with a midwife or an obstetrician, or both—involves conducting an in-depth investigation and assessment of the woman’s situation as well as planning, and that no decisions about the mode of delivery are made at this point. For example, in one of the guideline documents it was noted that during the first meeting the woman should be informed that, in most cases, the goal is a vaginal birth. In other guideline documents it was mentioned that it is important that the woman knows that she has been referred to the specialized maternity clinic to get help with handling her fears, and that she is informed that the process of deciding on the mode of delivery ‘*needs to take time’*. In one document it is expressed that it is important to show women with FOB that their fear is taken seriously, but without promising a CS. In several guideline documents it was explicitly stated that, without medical indications, CS is most often associated with a higher short- and long-term risk for both mother and child, and that the goal in most cases is therefore vaginal birth.

The following is an example of the process at one of the larger maternity clinics: When a CS is requested, the midwife at the antenatal clinic assesses whether FOB is the reason for this, and if so, the level of this fear. Basic information about both modes of delivery is given to the patient. If the request stands, an appointment with an obstetrician is booked. If the request still stands after this appointment, the midwife responsible for the patient, or the obstetrician, refers the patient to the clinic for women with FOB, in order to determine the mode of delivery.

There were also a few guideline documents that focus only on elective CS. Some of them described pre-op procedures such as who needs to see an anaesthesiologist beforehand, how health declarations are to be filled in, documentation in medical records, etc. These documents commonly specified which indications can be used as a basis for *any* physician to refer a patient to an elective CS, and in which cases *only* physicians in certain functions can do so (see more in the next section).

### Unclear how important factors are weighed in the decision-making

In more than half of the guideline documents, it was specified who makes the decision regarding mode of delivery when the woman has requested a CS. The woman’s role in the decision is not specified. One typical approach was that it is decided by an obstetrician at the specialized maternity clinic, while another is that it is decided by an obstetrician at the specialist maternity clinic in a specific forum, sometimes referred to as a section team. At the same time, in relatively many of the documents it was not specified who makes the decision, and only three documents specified when the decision is made (4–6 weeks before due date). The following is an example from one of the large delivery clinics: ‘*The obstetrician gives in-depth information about the medical risks and disadvantages involved with caesarean section. The antenatal midwife and obstetrician inform the patient that the decision about mode of delivery is made by a specific team, whereby the default plan is a vaginal birth/birth contract [a vaginal delivery is started but can be changed to a CS if the woman wants to] if there are no medical indications. Planned CS is only performed in exceptional cases towards the end of the pregnancy*.’ In another document it was specified that the final planning/decision regarding mode of delivery is made once a week during an obstetric round at the hospital, ‘*whereupon the woman is contacted to be informed of the decision*’.

Descriptions of how the decision is made varied. A number of documents listed factors that are relevant to consider when deciding on a CS (the same as in the national document; i.e. FOB, previous CS, previous birth injury, sexual abuse, previous stillborn or injured infant related to pregnancy or delivery, psychiatric illness, planning reasons). However, it was not specified in the documents how these factors should be interpreted or assessed when accommodating or rejecting a woman’s wish for CS. One example of a formulation was that a decision is made after ‘*a comprehensive assessment of whether the woman’s psychological wellbeing is an indication of CSMR’*; however, it is not specified on what grounds this assessment is to be made.

Four guideline documents specified what indications can be used as a basis for any physician to refer a woman to elective CS. In one document, these indications were: turning a breech baby to head failure; two or more earlier CSs; pelvic disproportion and previous elective or emergency CS; earlier sphincter rupture and remaining problems of significance; total or partial placenta praevia; colectomized patient with pelvic reservoir; duplex pregnancy with breech position on Twin 1; and earlier abdominal myomectomy. In the same guideline document, it was specified that only an obstetrician at the specialized maternity clinic can decide on CS based on non-medical indications, primary or secondary FOB (including those with previous complicated births), and physical or psychiatric illness with a physician without obstetric competence having recommended CS. In this document it was also noted that previous severe injury is not a reason for CS, but as it may cause FOB and hence a wish for CS among these women, they must be given additional time to discuss their injury and how to prevent it from happening again.

The most comprehensive local guideline document reiterated the conditions specified in the national indications: (i) patient-related conditions and (ii) care process-related conditions. The same factors that are of importance in the national guideline document were mentioned (e.g. age and previous birth injuries), but ethical aspects, the woman’s autonomy, equity aspects, professional values and responsibility, and health economic aspects are mentioned as well (also discussed in the national guideline document). However, there was no specification as to how to value the different factors, and taken together, the document signals a restrictive approach in which relevant factors do not include age (although aspects associated with age can be included), previous CS, previous severe birth injuries, or a previous stillborn or injured infant related to pregnancy or delivery (usually not included), but being a victim of sexual abuse or psychiatric illness can sometimes be considered a sufficient reason. It is pointed out, however, that individual assessments must be made.

Although most guideline documents implied a restrictive approach, a more permissive approach could occasionally be noted. For example, in one guideline document information about factors that can speak for ‘*a more liberal attitude*’ regarding the woman’s wish for a CS was earlier traumatic birth experiences and earlier emergency CS. In the national guideline document, it is specified that previous birth injuries, such as significant tearing and incontinence, are not a reason for CS unless there are *serious* and *persistent* problems. In line with this, some documents specified that a previous anal sphincter rupture with remaining problems of significance is a reason for a CS, while others only specified that there must be remaining problems, thus being more permissive. One guideline document also specified that in rare cases it can be necessary to promise a woman she will be able to have a CSMR in order for her to consider becoming pregnant. In two documents, it was mentioned that if the patient is convinced that CSMR is the best mode of delivery, even after receiving support and treatment, she will be given an elective CS. One example of this was that the decision to deny CSMR must be re-evaluated if the woman shows signs of a ‘strong and lasting crisis response’ even after receiving support and treatment. Overall, in some of the guidelines it was clearly stated that the woman (a possible partner is sometimes mentioned as well) should be involved in planning the care, in order to increase their participation. However, involvement in the actual CSMR decision was not stated in any of the documents.

### Birth contracts offered in some regions

In ten of the guideline documents, it was mentioned that a woman requesting a CS can be offered a ‘birth contract’, which typically means that she agrees to start a vaginal birth but can choose a CS during the delivery if she still wants it. In one guideline document it was expressed like this: ‘*Birth contracts or birth agreements can sometimes be an alternative to planned CS. This often applies to patients who initially requested a CS*.’ The same guideline document stated that these contracts ‘*have good results’*, but does not specify whether this means a lower rate of CSMR or greater satisfaction and sense of security for women giving birth. The sense of security was pointed out in another local guideline document: ‘*For these patients, the promise to be able to cancel is absolutely decisive for whether they dare to attempt vaginal birth, and must be respected’*.

In most guideline documents that mentioned birth contracts, a balance between the woman’s wish and the medical assessments was described. For example, in one of the guideline documents it was stated that the birth contract should be followed ‘*as far as the situation allows without jeopardizing medical safety’*. Furthermore, it was noted that it is important to give clear information on why the contract could not be followed, if this is the case, and that it is important to have dialogue and provide information afterwards. In two guideline documents it was noted that it is important to inform the patient that ‘*there are limitations to what can be promised beforehan*d’, and that it is always the physician responsible for all ongoing births at the time of birth who has the ultimate medical responsibility and thus the possibility to determine what is medically justifiable and to prioritize medical resources.

### The postpartum care is related to FOB rather than CSMR

In the guideline documents specifying postpartum care, it was related to FOB rather than the CS. It was suggested what kind of care should be offered, for instance information to child healthcare centres for follow-up, as FOB increases the risk of depression and feeding problems. Others suggested offering a telephone call during which the birth experience is rated and, based on the results of this, offering various kinds of care. Another example involved offering early follow-up visits to go over the birth experience, which is to be rated based on a visual analogical scale from 1 to 10. This is done at the postpartum check up at eight to twelve weeks. Among those with low ratings (1–5), the reasons for the low rating are discussed with the woman or the couple. It was also emphasized that women with extensive FOB should receive support already at the after-delivery ward and that the midwife at the antenatal clinic should be contacted and will then contact the woman within two weeks. One guideline document read: ‘*It is extra important to follow up on the birth experience for these women* [with FOB] —*already at the after-delivery ward with the visual analogical scale. If > 8* [FOB], *inform the midwife at the antenatal clinic, who will contact the patient within two weeks. If everything seems fine at that point, bring up the birth experience at the follow-up visit at the antenatal clinic’.*

Referral to a clinic for women with FOB should be considered if the birth experience is rated low. In one of the local guideline documents, it was expressed like this: ‘*It is extra important to have a follow-up visit for these women*—*already the first week if needed, but if everything seems okay by then, the birth experience should be brought up at the follow-up visit at the antenatal clinic or at the specialist antenatal clinic. Women with FOB have a higher risk of developing a negative birth experience and developing post-traumatic stress disorder. Feedback should be given to staff at delivery wards after good efforts, but also when a realistic birth plan has not been followed’*.

Some specialist clinics offer follow-up appointments, either by telephone or at physical meetings, whereas others leave the initiative for booking follow-up appointments to the woman/couple. In some documents it was stated when this follow-up should be done, for example two to three weeks postpartum. Others said to contact the woman during the first week postpartum for a first check up, and to conduct another one at the postpartum check up six to ten weeks after birth. There was also a description of possible long-term consequences should the follow-up not be conducted, such as developing FOB and post-traumatic stress syndrome.

## Discussion

In this study, we analysed local guideline documents on CSMR from all maternity clinics in Sweden that have them (32 out of 43 clinics). In summary, many of these documents framed women’s wish for a CS (i.e. elective CS without a medical indication) as a matter of FOB, and presented how women with different degrees of FOB should receive support and care in order to overcome their fear of vaginal birth, or at least increase their sense of security when giving birth vaginally. Before any decisions on CSMR are made, women with FOB are to take part in counselling. In many guideline documents it was explicitly stated that the goal is vaginal birth, and in some of them this was linked to CS implying greater short- and long-term risks to both mother and child, when medical indications are lacking. In general, the local guideline documents conveyed a restrictive approach to CSMR, but some of them included birth contracts; i.e., when a vaginal birth is started but can be converted into a CS if the woman wants this. It was pointed out, however, that this requires that the CS be safe from a medical point of view and that it not cause more urgent cases to be down prioritized.

Furthermore, many (but not all) guideline documents specified *who* makes the decision about the mode of delivery, usually either an obstetrician at a specialized maternity clinic or a caesarean section team. It was seldom specified *when* the decision was to be made. Although most guideline documents listed factors that are relevant to consider when making the decision (generally the same ones as in the national guidance document), they did not specify *how* to assess or interpret some of these factors. For example, how are *remaining (serious) problems after a birth injury* to be assessed, and by whom? Are they assessed based on physical or medical examination and evidence, or the woman’s experience from living with the injury? Are effects on the woman’s psychological well-being considered? Further, how is a *previous traumatic delivery* defined? Is this assessed based on the woman’s experience and lingering sense of trauma, or is it medically defined? These are questions that should be addressed when updating the national medical indications from 2011 as we believe this would reduce the risk of arbitrariness, which could also reduce local variation.

According to previous research, guideline documents developed for healthcare praxis can suffer from three kinds of problems [14]. First, the *interpretation problem* means that no matter how detailed guidelines are, they must always be interpreted by an individual. The interpretation problem is likely rather extensive in relation to some of the local guideline documents we examined, which were not very comprehensive, but is relevant to all guideline documents when it comes to *how* factors of potential importance should be assessed and weighed against each other. This opens for both individual differences among health professionals and differences among maternity clinics. Individual differences have been noted in previous research, and have been linked to health professionals’ personal preferences [15]. However, as one prominent challenge today in Sweden involves differing opinions on adequate reasons for CSMR between health professionals and women advocating for women’s rights to decide their mode of delivery, the interpretation problem could be reduced by specifying how assessments should be made, and not least how women’s subjective experiences should be weighed against medical examinations or evidence.

Second, the *multiplicity problem* means that there are a great number of guideline documents (parallel to laws and other regulations), which can pull in different directions. When it comes to CSMR, it is noteworthy that all the local guideline documents discussed here lacked reference to the Patient Law (2014:821), which stipulates that the patient’s autonomy and integrity shall be respected (Chap. 4, 1§) and that healthcare shall, as far as possible, be formulated and carried out in consultation with the patient (Chap. 5, 1§). CSMR can be understood as an expression of autonomy, and respect for autonomy is a central value in many countries’ healthcare practices. In healthcare a patient can exercise her autonomy through the process of informed consent, which implies that the patient should be properly informed about possible treatments for her condition and can then accept or decline the suggested treatment. In the case of CSMR, it is important to note that professional, beneficence-based clinical judgement means that respect for autonomy does not automatically mean that CS is offered [15]. However, to avoid paternalism, the requirement of deliberation, patient participation, and shared decision-making—the cornerstones of person-centred care—has emerged. This approach takes the stance that a person is characterized by rationality and thus deserves a special moral status that she can claim for herself and acknowledge in others [16]. There are disadvantages to not taking a person-centred or holistic perspective; Morgan and Yoder (2012) [17] argue that care ‘*that focuses on biological illness without considering the psychological or social impact hampers healing and contributes to poor outcomes’* (p. 8). Although pregnancy is not an illness, this reasoning should also be highly relevant when it comes to CSMR. In fact, proponents of CSMR argue that the evidence on the short- and long-term effects of vaginal birth vs. elective CS only involves biological aspects and does not include psychological effects from being denied one’s preferred mode of delivery, and, furthermore, lacks estimations of what it means to live with, e.g., urinal or anal incontinence and an inability to enjoy sex [9]. However, while person-centred care does not mean that the healthcare provider submits to doing everything a patient or pregnant woman wants, it is crucial to explore in-depth and try to understand the reasons behind a request for CS and to include the woman as far as possible in the process of deciding on the mode of delivery. Looking at the current local guideline documents in Sweden, it seems as if the decision of whether or not to perform a CSMR is currently not made in cooperation with the patient, but rather without the patient being present; e.g. among caesarean section teams. This is likely to cause feelings of exclusion among women who want a CS. Eide and Baerøe [18] argue that the relationship between a pregnant woman and the healthcare professional constitutes a moral relationship, with mutual rights and obligations. Therefore, it is not only the professional’s integrity and knowledge but also the woman’s ability to deliberate that should be respected. In the consent process, the woman must be informed of the short- and long-term health complications and risks involved with CS for both mother and child, as well as the fact that these risks can increase with repeated CS. Hence, even future pregnancies need to be considered in the decision process. At the same time, the woman must be met with respect, and the power imbalance between her and the healthcare provider must be handled responsibly. In Sweden, this process could be developed by using e.g. the Ottawa Decision Support Framework to assess decisional needs, provide decision support and evaluate decision outcomes [19]. It is likely to be hampered by the lack of staff. Understaffing of midwives is a national problem and one can speculate that requests for CSMR may increase even further as pregnant women know that shortage of midwives may lead to a more traumatic birth experience. With a planned CS, the pregnant women know beforehand that a midwife is reserved for the delivery. A study from Italy shows that severe understaffing in midwives’ work settings led to important underuse of standard protocols according to the international guidelines [20]. It could very well be the situation in Sweden as well and is an important aspect to consider when evaluating the increased demand for CSMR.

Third, the *legalization problem* means that guideline documents have come to be set up in increasingly legal form, more and more blurring the line between recommendations and legal obligations. Thereby, the focus can be moved from a reflection on what the morally best thing to do in a certain situation is to what is legally permitted. In this case, there is a risk that questions of, for example, patient autonomy and shared decision-making will be observed less, as the healthcare professional is focused on the normative parts of the local guidelines, leaning on current medical evidence on CSMR in Sweden. However, this is part of a larger discussion on the patient’s position in the Swedish healthcare system [21, 22]. Paradoxically, the Swedish welfare state is generous with social rights such as healthcare, but citizens have few legal rights to demand a service or to contest a particular choice of treatment or its timing. Nevertheless, the opportunities for choice have improved in recent decades, although this rather involves the choice of healthcare provider instead of the type of medical treatment or care. The Patient Law specifies that when there are several treatment or care options that are consistent with science and proven experience, the patient shall have the opportunity to choose the preferred option (if it appears to be justified, taking into account the illness or injury in question and the treatment costs). However, in the Swedish context, CS without medical indication is not considered an intervention consistent with either science or proven experience, as it is concluded that it implies a general increased risk for short- and long-term consequences for both mother and child, compared with vaginal birth [8]. It should be pointed out, that an adequate number of resources is necessary to implement new guidelines successfully. Both in terms of offering satisfactory care to the women but also as a guarantee for patient safety. The organizational conditions in Sweden, with a fairly limited number of clinics, should enable implementation of commonly decided national guidelines.

In sum, based on the local guideline documents, which to varying extents refer to the national guidance document, it is not surprising that the rate of CSMR in Sweden is low in international comparison. Overall, the local guideline documents convey a restrictive approach, which is likely impacting CSMR practice and Sweden’s relatively low level of CS. Furthermore, as they vary in content and comprehensiveness, it is also not surprising that CS rates vary between the Swedish regions. The lack of details, when it comes to how factors of potential importance for a CSMR should be assessed and weighed against each other, is likely to cause unnecessary practice variations as well as dissatisfaction among women who notice differences both between how individual health professionals interpret these factors and between regions. Furthermore, these practice variations do not meet the Swedish Healthcare Act’s (2017:30) requirements of good and equal care for all citizens, and there is thus a need for updated national guidance to improve local practice, potentially national guidelines. A knowledge compilation regarding elective CS from the National Board of Health and Welfare in 2022 did not contain any recommendations but continues a step in developing national guidelines for maternity care. It is stated the more studies within CSMR are needed. Both in terms of complications but also the reasons behind the discrepancy in experiences between health care professionals and women requesting CS. Knowledge we aim to contribute with. The first national guidelines for maternity/delivery care are however planned to be released by the National Board of Health and Welfare at the end of 2023. Whether guidelines regarding CSMR is included in this document remains to be seen.

### Strengths and limitations

A major strength with this study is that all maternity clinics in Sweden were included, regardless of whether they had local guideline documents for CSMR. Despite this, a limitation was the different kinds of guideline documents, which made the analysis somewhat challenging. However, after reading the guideline documents several times and performing the coding, a clearer pattern could be identified. To strengthen credibility, triangulation was used. Two authors analysed the material, first separately and then together, which resulted in multiple observations and conclusions. This type of triangulation was thought to provide both a confirmation of findings and different perspectives, adding breadth to the study.

The Framework Method was used as it offers a clear step-by-step process, which is suitable for interdisciplinary and collaborative studies, as in this case. The authors have different professions, experience, and backgrounds, but with the important common ground of conducting research within healthcare. As previously stated, CSMR is a complex issue within healthcare, and in order to create a cohesive national guideline document, a multifaceted approach must be taken. As all maternity clinics in Sweden participated, transferability is possible.

## Conclusions

To offer women who request CS equal and just care, there is a pressing need to either adhere to the current national medical indications at all maternity clinics or rewrite the various local guideline documents to enable the clinics to adopt a structured approach. Emphasis must be placed on exploring the reasons behind the request for CS and providing unbiased information and support. All discussions should comply with the principles of medical ethics and include all benefits and risks, to both woman and child. It is important not only to address women’s concerns and offer counselling for FOB but also to understand that there may be many reasons besides FOB for requesting a CS. One such reason is bodily autonomy. Regardless of the reason for CSMR, we should enhance women’s capacity for autonomous choice, strengthen reproductive rights, and offer person-centred care based on shared decision-making. Hopefully, the results here will contribute to the ongoing discussion about CSMR and guidelines/recommendations and lay a foundation for further research in which professionals as well as stakeholders and women can give their views on this issue.

## Data Availability

The datasets used and/or analysed during the current study are available from the corresponding author on reasonable request.

## List of abbreviations

CS: Caesarean section
CSMR: Caesarean section on maternal request
FOB: Fear of birth
